# Genome-wide Association Analysis of Eye Movement Dysfunction in Schizophrenia

**DOI:** 10.1038/s41598-018-30646-9

**Published:** 2018-08-17

**Authors:** Masataka Kikuchi, Kenichiro Miura, Kentaro Morita, Hidenaga Yamamori, Michiko Fujimoto, Masashi Ikeda, Yuka Yasuda, Akihiro Nakaya, Ryota Hashimoto

**Affiliations:** 1 Department of Genome Informatics, Graduate School of Medicine, Osaka University, Osaka, Japan; 2 Department of Integrative Brain Science, Graduate School of Medicine, Kyoto University, Kyoto, Japan; 3 Department of Neuropsychiatry, Graduate School of Medicine, The University of Tokyo, Tokyo, Japan; 4 Department of Psychiatry, Graduate School of Medicine, Osaka University, Osaka, Japan; 5 Department of Psychiatry, Fujita Health University School of Medicine, Aichi, Japan; 6 Molecular Research Center for Children’s Mental Development, United Graduate School of Child Development, Osaka University, Osaka, Japan; 7 Department of Pathology of Mental Diseases, National Institute of Mental Health, National Center of Neurology and Psychiatry, Tokyo, Japan

## Abstract

Eye movements are considered endophenotypes of schizophrenia. However, the genetic factors underlying eye movement are largely unknown. In this study, we explored the susceptibility loci for four eye movement scores: the scanpath length during the free viewing test (SPL), the horizontal position gain during the fast Lissajous paradigm of the smooth pursuit test (HPG), the duration of fixations during the far distractor paradigm of the fixation stability test (DF) and the integrated eye movement score of those three scores (EMS). We found 16 SNPs relevant to the HPG that were located in 3 genomic regions (1q21.3, 7p12.1 and 20q13.12) in the patient group; however, these SNPs were intronic or intergenic SNPs. To determine whether these SNPs occur in functional non-coding regions (*i*.*e*., enhancer or promoter regions), we examined the chromatin status on the basis of publicly available epigenomic data from 127 tissues or cell lines. This analysis suggested that the SNPs on 1q21.3 and 20q13.12 are in enhancer or promoter regions. Moreover, we performed an analysis of expression quantitative trait loci (eQTL) in human brain tissues using a public database. Finally, we identified significant eQTL effects for all of the SNPs at 1q21.3 and 20q13.12 in particular brain regions.

## Introduction

Schizophrenia is a psychiatric disease with a lifetime prevalence of 0.30–0.66%^1^. The diagnostic criteria for schizophrenia are based on subjective symptoms, including delusions, hallucinations and thought insertion, and objective symptoms, including disorganized speech and bewilderment. The age of onset is usually late adolescence. Nevertheless, it is difficult to diagnose schizophrenia in patients in late adolescence on the basis of clinical symptoms alone^2,3^. Although the development of a quantitative biomarker to aid in diagnosis is an attractive strategy, no such markers have been identified. Neuropsychological studies have shown that eye movement dysfunction is a clinical symptom of schizophrenia. Abnormalities in smooth pursuit eye movements (SPEMs)^4–6^, voluntary control of saccades^7–9^ and exploratory eye movement behaviors^10–13^ have been commonly observed. Studies comparing eye movement scores between non-schizophrenic individuals and patients with schizophrenia have distinguished the groups with accuracies of 80–90%^14–17^.

Several studies have reported that eye movement performance is limited not only in patients with schizophrenia but also in their siblings^18,19^, thus suggesting that eye movements may be a candidate endophenotype of schizophrenia. The heritability of smooth pursuit and anti-saccadic eye movement tasks was estimated as 0.4 to 0.6 in a family-based analysis of schizophrenia^20,21^. In particular, predictive pursuit gain in the smooth pursuit test has a very high heritability (heritability = 0.9)^22^, indicating that genetic factors underlie abnormalities in eye movement. However, it remains unknown whether patients with schizophrenia are predisposed to dysfunctional eye movements.

Genomic analyses have been performed to identify susceptibility loci for eye movement. The genetic loci related to SPEMs have been observed in the regions surrounding ERBB4^23^, RANBP1^24^, COMT^25^, ZDHHC8^26^ and NRG1^27^. In EEM, the eye fixations for the responsive search and cognitive search scores are associated with chromosomes 22q11.2^28^ and 5q21.3^29^, respectively. However, most previous studies focused on only specific gene polymorphisms. To extensively search for susceptibility loci in genomes, a genome-wide association study (GWAS) is more effective.

In this study, we performed a GWAS to identify SNPs associated with the following eye movement scores: the scanpath length during the free viewing test (SPL); the horizontal position gain during the fast Lissajous paradigm of the smooth pursuit test (HPG); and the duration of fixations during the far distractor paradigm of the fixation stability test (DF). Our previous study revealed that these eye movement scores distinguish patients with schizophrenia^17^. Our GWAS also included the integrated eye movement score (EMS), composed of these three measures, which showed discrimination ability with an accuracy of 82%. Our analyses revealed 17 susceptibility loci for HPG. Furthermore, chromatin state analysis showed that the SNPs at 1q21.3 and 20q13.12 are located in enhancer or promoter regions. The SNPs at 1q21.3 were located in intron or intergenic regions of the THEM4 or S100A10 genes, and the SNP at 20q13.12 was located upstream of the CDH22 gene. THEM4 is a negative regulator of the AKT1 gene, contributing to signal transduction in neurons^30^. S100A10 expression is lower in lymphoblastoid cell lines from patients with schizophrenia than in those from controls^31^. Cadherins such as CDH22 are involved in synaptic plasticity, which may contribute to learning and memory^32^. Recent studies have suggested that synaptic plasticity dysfunction is involved in schizophrenia^33,34^. To examine the functions of these SNPs in detail, our bioinformatics analyses showed that these SNPs were located in functional non-coding regions, such as enhancers or promoters, and affect the expression level of surrounding genes in specific brain regions.

## Materials and Methods

### Subjects

Eye movements were recorded in 60 patients with schizophrenia and 166 healthy control participants who were recruited at Osaka University. These subjects were included in previous studies^35–39^. All of the subjects were of Japanese descent and were biologically unrelated. They had no history of ophthalmologic disease or neurological/medical conditions that influence the central nervous system^40,41^. Specific exclusion criteria included atypical headaches, head trauma with loss of consciousness, chronic lung disease, kidney disease, chronic hepatic disease, thyroid disease, active cancer, cerebrovascular disease, epilepsy, seizures, substance-related disorders, and mental retardation. Healthy control participants were recruited through regional advertisements at Osaka University and were evaluated for psychiatric, medical, and neurological concerns by using the non-patient version of the Structured Clinical Interview for DSM-IV (SCID) to exclude individuals with current or past contact with psychiatric services and those who had received psychiatric medication.

Patients with schizophrenia were recruited from Osaka University Hospital and had been diagnosed by two or more trained psychiatrists according to the DSM-IV criteria on the basis of the SCID. The current symptoms of schizophrenia were evaluated. The total dose of their prescribed antipsychotics was calculated in chlorpromazine (CPZ) equivalents (mg/day)^42^. The current IQ was measured using the Japanese version of the Wechsler Adult Intelligence Scale-Third Edition (WAIS-III)^43^. Premorbid IQ was estimated using the Japanese version of the National Adult Reading Test^44,45^. The study was performed in accordance with the World Medical Association’s Declaration of Helsinki and was approved by the Research Ethics Committee of Osaka University. All of the participants provided written informed consent to participate in the study after a full explanation of the study procedures was provided. Anonymity was preserved for all participants.

### Measurement of eye movement

The subjects faced a 19-in liquid crystal display monitor placed at a 70-cm distance from the observers’ eyes. Visual stimuli were presented using MATLAB (MathWorks, Natick, MA, USA) via the Psychophysics Toolbox extension^46^. The eye movements and pupil areas of the left eye were measured at 1 kHz using the EyeLink1000 (SR Research, Ontario, Canada) system. Measurements of the SPL, HPG and DF were collected as previously described^16,17^. The integrated EMS of the above three scores was calculated as in Morita *et al*.^17^.

### Genotyping

Genotyping was performed using the Affymetrix Genome-Wide Human SNP Array 6.0 (Affymetrix, Santa Clara, CA, USA) according to the manufacturer’s protocol. The genotypes were called from the CEL files by using Birdseed v2 for the 6.0 chip implemented in the Genotyping Console software (Affymetrix). We then applied the following QC criteria to exclude samples: (i) arrays with low QC (<0.4) according to Birdseed v2 (n = 0), (ii) samples for which <90% of the genotypes were called (n = 0) and (iii) samples in the same family according to pi-hat (>0.1, n = 0). Next, we excluded SNPs that (i) had low call rates (<0.97), (ii) were duplicated, (iii) were located on sex chromosomes, (iv) deviated from HWE in the controls (p < 1.0 × 10^−5^) or (v) had low MAF <0.01. After these QC exclusions, 554, 152 SNPs were retained for experimental analysis. Of the 554, 152 SNPs, we used SNPs for which the heterozygous genotype and homozygous minor allele genotype were present in more than 3 subjects for each measurement in each group. To test for the existence of genetic structure in the data, we performed a principal component analysis (PCA) using the EIGENSTRAT 3.0 software^47^. Twenty eigenvectors were calculated. Genotype information from the JPT (Japanese in Tokyo, Japan), CHB (Han Chinese in Beijing, China), CEU (Utah residents with ancestors from northern and western Europe) and YRI (Yoruba in Ibadan, Nigeria) in HapMap 3 was compared with our dataset to check for population stratification. Genotyping was performed as described in previous studies^35–39^.

### Statistical analysis

A t-test was performed to measure statistical significance for all variables except sex by using R 3.3.0. Statistical significance for sex was calculated with the χ^2^ test. We performed linear regression analysis to identify SNP markers associated with the four eye movement scores using PLINK version 1.07^48^.

### Analysis of functional non-coding regions

Functional non-coding regions, including enhancer and promoter regions, were searched by using the chromatin segmentation data from ChromImpute, which predicts chromatin states from a mathematical model based on the imputed data for 11 histone modifications and DNase from the Roadmap Epigenomics Project^49,50^. We used the chromatin segmentation data for 127 human cell lines and tissues (http://epigenomegateway.wustl.edu/browser/).

### Cis-expression QTL (eQTL) analysis in brain tissues

We used the BRAINEAC database to perform cis-eQTL analysis in brain regions^51^. BRAINEAC is a database that includes SNPs associated with gene expression from 134 individuals in ten post-mortem brain regions: cerebellar cortex, frontal cortex, hippocampus, inferior olivary nucleus (sub-dissected from the medulla), occipital cortex, putamen (at the level of the anterior commissure), substantia nigra, temporal cortex, thalamus (at the level of the lateral geniculate nucleus) and intralobular white matter. The eQTL analysis used linear regression to delineate the effects of genotype against the expression level of the genes closest to the SNPs.

## Results

### Comparison of demographic data between healthy control participants and patients with schizophrenia

Our analyses included 226 subjects (60 patients with schizophrenia (SZ group) and 166 healthy control participants (HC group)). Participant demographics are provided in Table 1. The HC and SZ groups were not significantly different in sex (p = 0.280) but were significantly different in age (p = 2.46 × 10^−4^). We used the three eye movement scores reported in our previous study that best distinguished between healthy controls and schizophrenic individuals^17^. As expected, the three eye movement scores for the HC group were significantly higher than those for the SZ group (SPL: p = 2.78 × 10^−13^; HPG: p = 1.61 × 10^−7^; DF: p = 1.92 × 10^−5^). Similarly, the EMS of the HC group was also significantly higher than that of the SZ group (p = 2.20 × 10^−16^).

**Table 1:** Statistical summary of individuals in healthy control (HC) and schizophrenia (SZ) datasets.

	HC dataset (N = 166)	SZ dataset (N = 60)	P-value (t-test or χ^2^ test)
Sex (Male/Female)	96/70	29/31	0.280
Age (years)	28.6 ± 11.7	34.9 ± 10.9	2.46 × 10^−4^
SPL	109.7 ± 24.6	73.4 ± 29.4	2.78 × 10^−13^
HPG	1.07 ± 0.052	1.01 ± 0.079	1.61 × 10^−7^
DF	2267.8 ± 1444.6	1461.2 ± 1110.6	1.92 × 10^−5^
EMS	0.378 ± 0.842	−1.27 ± 1.12	2.20 × 10^−16^

SPL: the scan path length during the free viewing test; HPG: the horizontal position gain during the fast Lissajous paradigm of the smooth pursuit test; DF: the duration of fixations during the far distractor paradigm of the fixation stability test; EMS: the integrated eye movement score using three measurements (SPL, HPG and DF).

### Whole-genome QTL analysis of eye movement scores

**Figure 1:**
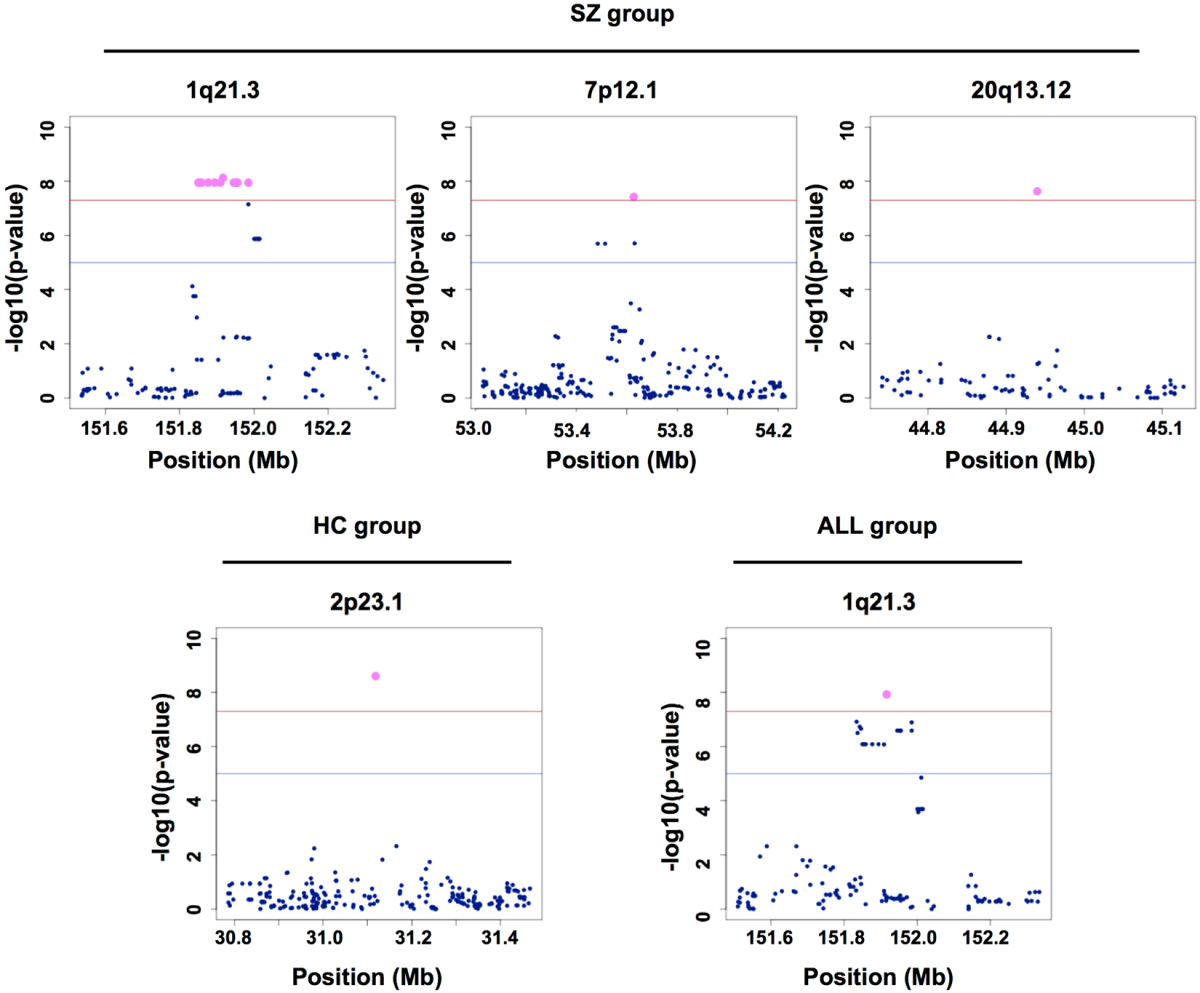
Genetic mapping in each group. The red line and blue line indicate a genome-wide significant p-value (p = 5.0 × 10^−8^) and a suggestive p-value (p = 1.0 × 10^−5^), respectively. The magenta plot indicates that a SNP marker satisfies a genome-wide significant p-value (p = 5.0 × 10^−8^).

To search for SNP markers associated with the three eye movement scores and the integrated score, we performed a GWAS by using linear regression analysis in each group and in all of the samples (SZ, HC and ALL (the combined SZ and HC groups)). We found that several SNP markers yielded a genome-wide significant p-value (p = 5.0 × 10^−8^) for HPG in each group (1 SNP in the HC group, 16 SNPs in the SZ group and 1 SNP in the ALL group) (Fig. 1, Table 2 and Fig. S1), although there were no significant SNP markers for the other measurements (Figs S2–4). In the HC group, we found a SNP marker, rs17393065, that is located 15 kb 3′ of GALNT14. In the SZ group, we found 16 SNPs in 3 genomic regions (1q21.3, 7p12.1 and 20q13.12). Of the 14 SNPs on 1q21.3, 4 SNPs are in a THEM4 intron, and 10 SNPs are in an intergenic region between THEM4 and S100A10. The SNPs on 7p12.1 and 20q13.12 were 523 kb 3′ of POM121L12 and 2.6 kb 5′ of CDH22, respectively. In the ALL group, we found a SNP marker, rs1490191, located 34 kb 5′ of THEM4. Interestingly, this SNP marker was also significantly associated with HPG in the SZ group. The number of major alleles for all 18 identified SNPs was positively correlated with HPG, such that as the number of minor alleles increased, HPG decreased. To control for a significant difference between the HC and SZ groups in terms of age, we adjusted for the effect of age by linear regression analysis with age as an independent variable. Consequently, the 17 SNPs, except for one SNP at 7p12.1 in the SZ group, remained statistically significant.

**Table 2:** Whole-genome QTL results with the horizontal position gain (HPG).

SNP ID	Cytogenic location	Closest gene	Location	Beta	P-value	P-value (age-controlled)
**SZ group**						
rs6587628	1q21.3	THEM4	intron	−0.1673	1.11E-08	2.79E-08
rs4845388	1q21.3	THEM4	intron	−0.1673	1.11E-08	2.79E-08
rs6685291	1q21.3	THEM4	intron	−0.1673	1.11E-08	2.79E-08
rs6691350	1q21.3	THEM4	intron	−0.1673	1.11E-08	2.79E-08
rs1552606	1q21.3	THEM4	intergenic	−0.1673	1.11E-08	2.79E-08
rs3007682	1q21.3	THEM4	intergenic	−0.1673	1.11E-08	2.79E-08
rs1490191	1q21.3	THEM4	intergenic	−0.1379	7.40E-09	1.88E-08
rs3007700	1q21.3	S100A10	intergenic	−0.1673	1.11E-08	2.79E-08
rs2999525	1q21.3	S100A10	intergenic	−0.1673	1.11E-08	2.79E-08
rs2999529	1q21.3	S100A10	intergenic	−0.1673	1.11E-08	2.79E-08
rs1532132	1q21.3	S100A10	intergenic	−0.1673	1.11E-08	2.79E-08
rs1387835	1q21.3	S100A10	intergenic	−0.1673	1.11E-08	2.79E-08
rs1387834	1q21.3	S100A10	intergenic	−0.1673	1.11E-08	2.79E-08
rs2999538	1q21.3	S100A10	intergenic	−0.1673	1.11E-08	2.79E-08
rs6964854	7p12.1	POM121L12	intergenic	−0.182	3.73E-08	9.01E-08
rs6104543	20q13.12	CDH22	intergenic	−0.1984	2.34E-08	3.88E-08
**HC group**						
rs17393065	2p23.1	GALNT14	intergenic	−0.149	2.595E-09	1.79E-09
**ALL group (HC** + **SZ group)**						
rs1490191	1q21.3	THEM4	intergenic	−0.0934	7.03E-09	1.09E-08

Beta: regression coefficient; P-value: Wald test asymptotic p-value; P-value (age-controlled): p-value controlled by age.

### Location of susceptibility loci for eye movement in functional non-coding regions

**Figure 2:**
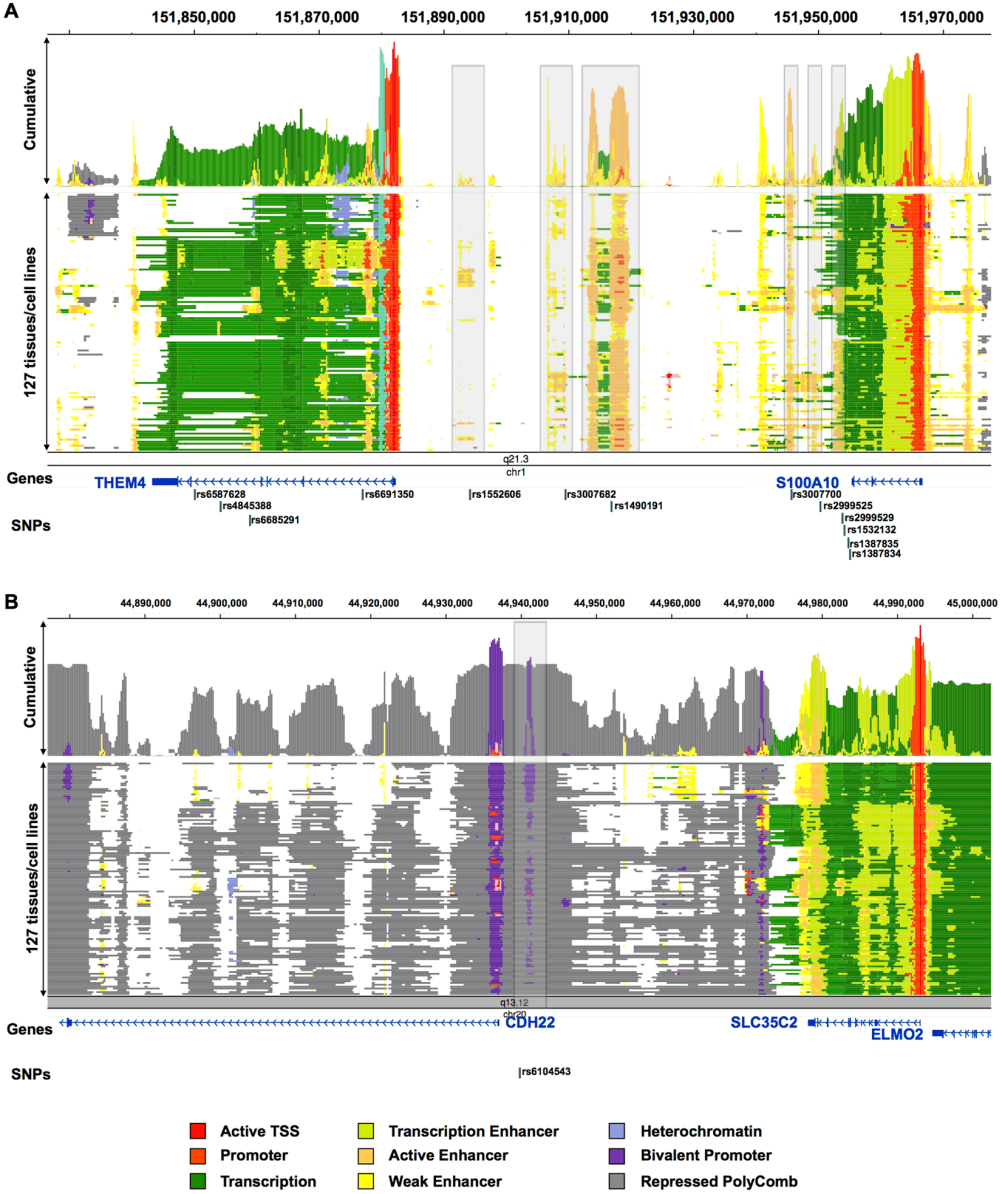
The state model of chromatin in 1q21.3 (**A**) and 20q13.12 (**B**). The chromatin was segmented into 25 states (*i*.*e*., promoter, enhancer) with the ChromHMM and ChromImpute algorithms^45,46^ by using data from the NIH Roadmap Epigenomics Consortium (http://www.roadmapepigenomics.org) and was visualized with the WashU Epigenome Browser (http://epigenomegateway.wustl.edu/browser/). Here, 25 states are summarized to 9 as shown in the color legend. The gray shaded stripe represents an active/weak enhancer (**A**) or bivalent promoter regions (**B**).

The SNPs found in this study were located in non-coding regions, such as introns and intergenic regions, which do not directly affect protein function. We examined the possibility of functional variants around identified SNPs. Most of the SNPs identified by GWAS do not affect the coding regions of genes. Functional variants may be in strong linkage disequilibrium (LD) with SNPs identified by GWAS. To identify variants in gene-coding regions genetically linked to the identified SNPs, we used the HaploReg database (Table S1). We extracted SNPs that were strongly linked to the identified SNPs based on the Asian population in the 1000 Genome Project (r^2^ > 0.8). Among them, we found 2 SNPs (rs3762427 and rs3748805) in the THEM4 coding region. rs3762427 and rs3748805 were synonymous and missense variants, respectively. To predict whether the missense variant rs3748805 is deleterious, we used three different programs (PolyPhen2, SIFT and PROVEAN). rs3748805 was “BENIGN” in the PolyPhen2 program, “Tolerated” in the SIFT program and “Neutral” in the PROVEAN program. These results showed that there were no functional variants in coding regions. Therefore, we next investigated the possibility that these SNPs may be located in functional non-coding regions (*i*.*e*., enhancers). To check them, we used the chromatin state from 127 human cell lines or tissues from the Roadmap Epigenomics Project. The chromatin states were predicted on the basis of 12 epigenomic markers (H3K4me1, H3K4me2, H3K4me3, H3K9ac, H3K27ac, H4K20me1, H3K79me2, H3K36me3, H3K9me3, H3K27me3, H2A.Z, and DNase) by using ChromHMM and ChromImpute^49,50^. The intergenic SNPs at 1q21.3 observed in the SZ group were near enhancer regions (yellow or orange segments in Fig. 2A). The SNP marker rs1490191, which was also identified in the ALL group, was in an active enhancer region (orange segments in Fig. 2A). These results suggested that the SNPs at 1q21.3 are located around enhancer regions and affect the expression of genes in the vicinity of the SNPs. We also found that the SNP rs6104543, upstream of CDH22 at 20q13.12, was located around bivalent promoters, which are regions that contain both a “repressive” and an “activating” chromatin modification^52^ (dark purple segments in Fig. 2B). We did not find functional non-coding regions around the other SNPs (7p12.1 in the SZ group; 2p23.1 in the HC group) (Fig. S5).

### eQTL effect of susceptibility loci for eye movement

As shown above, the SNPs at 1q21.3 and 20q13.12 found in the SZ and ALL groups were around the THEM4, S100A10 and CHD22 genes. These genes are associated with neurotransmission and brain morphogenesis. THEM4 (thioesterase superfamily member 4) is also known as CTMP (carboxyl-terminal modulator protein) and is a negative regulator of the AKT1 gene, contributing to signal transduction in neurons^30^. S100A10 (S100 calcium binding protein A10) modulates the transport of neurotransmitters (*e*.*g*., calcium ion, serotonin)^53^. CDH22 (cadherin 22) is mainly expressed in the brain and is involved in brain morphogenesis as a cell-adhesion factor^54,55^. These genes may be involved because eye movements are closely linked to the activity of specific brain regions^56–58^.

**Figure 3:**
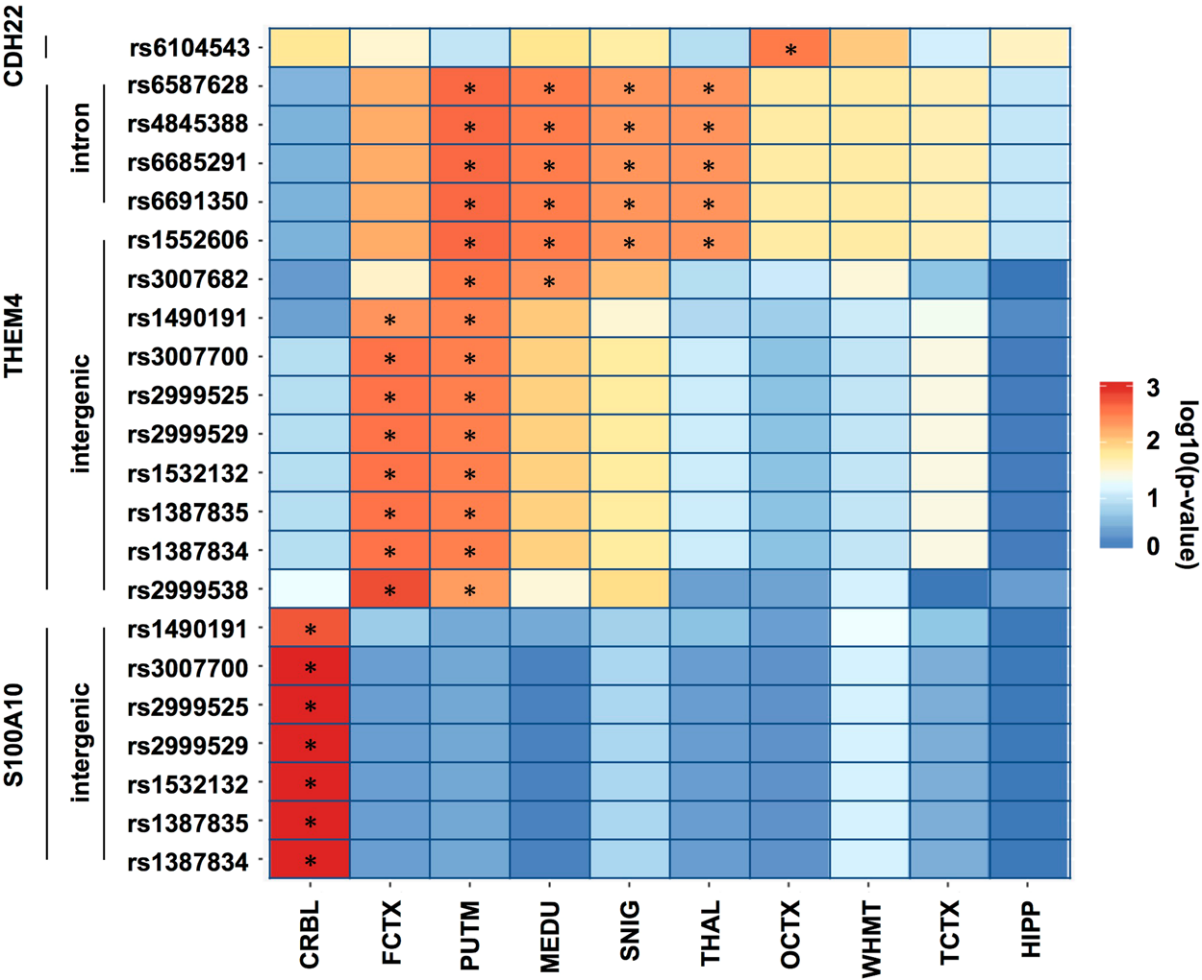
Heatmap based on statistics from the eQTL analysis. Each color represents the significance (log10 (p-value)) of the association between the genotype of a SNP and the expression level of the gene that is shown for each line. P-values were provided by the BRAINEAC database (http://www.braineac.org/). An asterisk indicates that a SNP satisfies a significance threshold for the Bonferroni correction (0.05/10 brain regions = 0.005) in a brain region. This plot displays only significant eQTL associations in at least one brain region. CRBL, cerebellar cortex; FCTX, frontal cortex; PUTM, putamen; MEDU, medulla; SNIG, substantia nigra; THAL, thalamus; OCTX, occipital cortex; WHMT, intralobular white matter; TCTX, temporal cortex; HIPP, hippocampus.

To further examine whether the SNPs at 1q21.3 and 20q13.12 are associated with the expression levels of the surrounding genes in brain regions, we analyzed eQTL by using the BRAINEAC database, including 10 human brain regions (cerebellar cortex (CRBL), frontal cortex (FCTX), hippocampus (HIPP), medulla (specifically the inferior olivary nucleus, MEDU), occipital cortex (specifically the primary visual cortex, OCTX), putamen (PUTM), substantia nigra (SNIG), thalamus (THAL), temporal cortex (TCTX) and intralobular white matter (WHMT)). The eQTL analysis used linear regression to delineate the effects of genotype against the expression level of genes around the SNPs. Consequently, all of the SNPs at 1q21.3 were found to be significantly associated with THEM4 and/or S100A10 expression in at least one brain region. The significance threshold for the Bonferroni correction was set at 0.005 (=0.05/10 brain regions). As expected, 4 intronic SNPs at THEM4 were significantly associated with THEM4 but not S100A10 expression (Fig. 3). These SNPs satisfied a significance threshold in 4 brain regions (PUTM, MEDU, SNIG and THAL). In contrast, 7 intergenic SNPs showed a significant eQTL association with THEM4 in 2 brain regions (FCTX and PUTM). PUTM was common to all SNPs associated with THEM4. Additionally, 7 intergenic SNPs were significantly associated with S100A10 in the CRBL. The rs6104543 upstream of CDH22 was significant for OCTX. These results suggested that SNPs at 1q21.3 and 20q13.12 act as cis-eQTL and that these effects are different in each brain region.

## Discussion

In this study, we explored susceptibility loci for three eye movement scores (SPL, HPG and DF) and the integrated eye movement score (EMS). We found 17 susceptibility loci for HPG in the SZ and ALL groups. Furthermore, our results showed that the SNPs at 1q21.3 and 20q13.12 are located in enhancer or promoter regions and that the genotypes of those SNPs are associated with the expression levels of the surrounding genes in specific brain regions.

To the best of our knowledge, our study is the first report using SNPs to explain the abnormal value of the horizontal position gain in the fast Lissajous paradigm of the smooth pursuit test in patients with schizophrenia. We found that the SNPs were only associated with HPG among four eye movement scores. Several studies have reported that SPEMs are inherited^18–21^. In particular, predictive pursuit gain, which is related to HPG, has very high heritability (heritability = 0.9)^22^. Therefore, we considered that the SNPs were associated with only HPG. In previous genomic studies of SPEMs, the natural logarithmic values of the signal/noise ratio (SNR)^23–26^ or velocity gain and saccadic frequency were used^27^ in the smooth pursuit test. In our study, we used the HPG as a measurement in the smooth pursuit test because our previous study showed that the HPG, rather than other measurements (*i*.*e*., SNR), has high discriminatory ability^17^. Our results may help elucidate the genetic association of eye movements from a different perspective in the smooth pursuit test.

We showed that the SNPs at 1q21.3 affect the expression of the THEM4 and S100A10 genes, which are near the SNP loci. THEM4 is a negative regulator of the AKT1 gene, which showed lower expression values in the frontal cortex of schizophrenic patients^59^. Our eQTL analyses showed that the SNPs on 1q21.3 were associated with the expression level of THEM4 in the frontal cortex, thus suggesting that THEM4 affects AKT1 expression in the frontal cortex. We have reported that the AKT1 genotype is related to gray matter volumes in schizophrenia patients in the frontostriatal region, which is part of the frontal cortex^60^. The frontal cortex contains the frontal eye field, an essential area for saccade and smooth pursuit eye movements^61–63^. The expression of THEM4 in the frontal cortex could be related to eye movements. We showed eQTL effects of S100A10 in the cerebellar cortex. S100A10 is up-regulated in Purkinje cells in a mouse model of inflammatory demyelinating diseases in the central nervous system^64^. The cerebellar cortex is involved in various motor activities and plays a role in smooth pursuit eye movement^65^. Moreover, we also identified CHD22 as the closest gene to a SNP on 20q13.12 in the SZ group. An intronic SNP on 20q13.12 was correlated with CDH22 expression in the occipital cortex. The occipital cortex is closely related to visual processing. V5, which is a region in the visual cortex, is responsible for the guidance of smooth pursuit, and lesions in V5 cause defects in smooth pursuit^66^.

We performed replication analysis for eye movement markers found in previous studies (Table S2). Consequently, 2 of 7 SNPs in the ERBB4, RANBP1, COMT and MAN2A1 genes showed significant associations with eye movement scores in our study (p < 0.05); however, these p-values were determined before correction for multiple tests. It is reasonable for some markers to be replicated in the Japanese population because these markers are also found in Korean and Chinese populations, which are genetically closely related to the Japanese population. Our results suggested that the effects of these markers on eye movement are constant, at least in East Asian populations.

Our study has several limitations. Our identified SNPs have small effect sizes. A power analysis based on α = 5 × 10^−8^ and 554, 152 SNPs estimated a power of approximately 0.2 for rs1490191 (MAF = 10% (dbSNP)) in the ALL group (n = 226). Although our study fortunately found some associated SNPs, our modest sample size likely resulted in our missing associations with other SNPs (type II error). To enhance the effect sizes and ensure the robustness of our findings, *i*.*e*., reduce type I error, we must collect more samples in an international multicenter study and must perform validation and replication. Another limitation is the significant difference between the HC and SZ groups in age (Table 1). Although we showed that the 17 SNPs, except for a SNP on 7p12.1 in the SZ group, remained statistically significant after linear regression analysis with age as an independent variable, analyses with age-matched controls are needed in future work. We suggested that three genes (THEM4, S100A10 and CDH22) are associated with eye movements. However, these associations are indirect. To support our results in future work, we need to identify whether the genes suggested in this study are associated with eye movements.

In conclusion, we have identified 17 potential susceptibility loci for HPG. Although these SNPs were found in non-coding regions, chromatin state analyses revealed that some of these SNPs were located in enhancer or promoter regions. Furthermore, eQTL analysis supported the finding that the SNPs in enhancer or promoter regions affected the expression levels of the surrounding genes. However, the sample size in our study is still too small to find established SNPs. In the future, an independent replication test should be performed to ensure the robustness of our findings.

## Electronic supplementary material

Supplement Figures

Table S1

Table S2

## Data Availability

The datasets generated during and/or analyzed during the current study are not publicly available because they contain information that could compromise research participant privacy/consent but are available from the corresponding author on reasonable request.
