# Supplementary material for: Genome-wide Association Analysis of Eye Movement Dysfunction in Schizophrenia
Source: Sci Rep. 2018 Aug 17;8:12347. doi: 10.1038/s41598-018-30646-9 (PMC6098030; doi:10.1038/s41598-018-30646-9)
Supplement: Supplementary file 1 — Supplement Figures [file 41598_2018_30646_MOESM1_ESM.pdf]

Genome-wide Association Analysis of Eye Movement Dysfunction in Schizophrenia

Masataka Kikuchi [a], Kenichiro Miura [b], Kentaro Morita [c], Hidenaga Yamamori [d], Michiko Fujimoto [d], Masashi Ikeda [e], Yuka Yasuda [d], Akihiro Nakaya [a], Ryota Hashimoto [d,f,g,*]

[a]Department of Genome Informatics, Graduate School of Medicine, Osaka University, Osaka, Japan
[b]Department of Integrative Brain Science, Graduate School of Medicine, Kyoto University, Kyoto, Japan
[c]Department of Neuropsychiatry, Graduate School of Medicine, The University of Tokyo, Tokyo, Japan
[d]Department of Psychiatry, Graduate School of Medicine, Osaka University, Osaka, Japan
[e]Department of Psychiatry, Fujita Health University School of Medicine, Aichi, Japan
[f]Molecular Research Center for Children's Mental Development, United Graduate School of Child Development, Osaka University, Osaka, Japan
[g]Department of Pathology of Mental Diseases, National Institute of Mental Health, National Center of Neurology and Psychiatry, Tokyo, Japan

*Corresponding author. Tel.: +81 6 6879 3074, Fax: +81 6 6879 3074
Molecular Research Center for Children's Mental Development, United Graduate School of Child Development, Osaka University, D3, 2-2, Yamadaoka, Suita, Osaka, 5650871, Japan;
E-mail: hashimor@psy.med.osaka-u.ac.jp (R. Hashimoto)

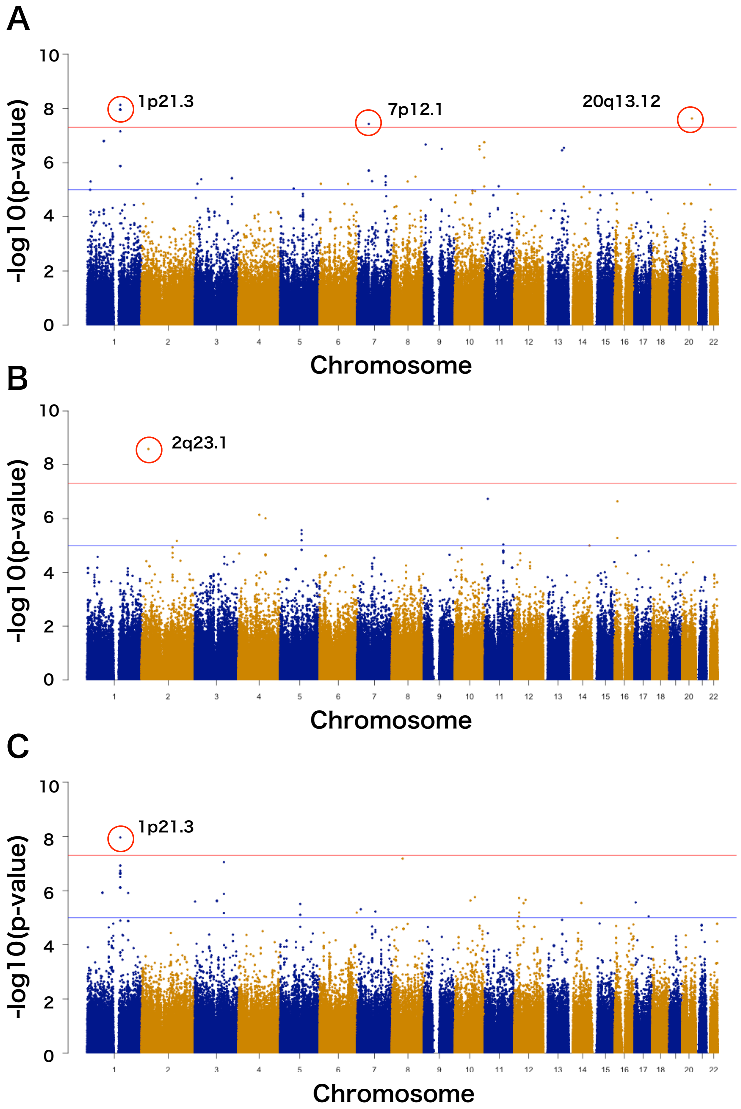

Figure S1. QTL analysis of HPG in schizophrenia (SZ), healthy control (HC) and ALL groups.
Manhattan plots in 60 SZ individuals (A), 166 HC individuals (B) and all 226 individuals (C). Red line and blue line indicate a genome-wide significant p-value ($p=5\times10^{-8}$) and a suggestive p-value ($p=1\times10^{-5}$), respectively.

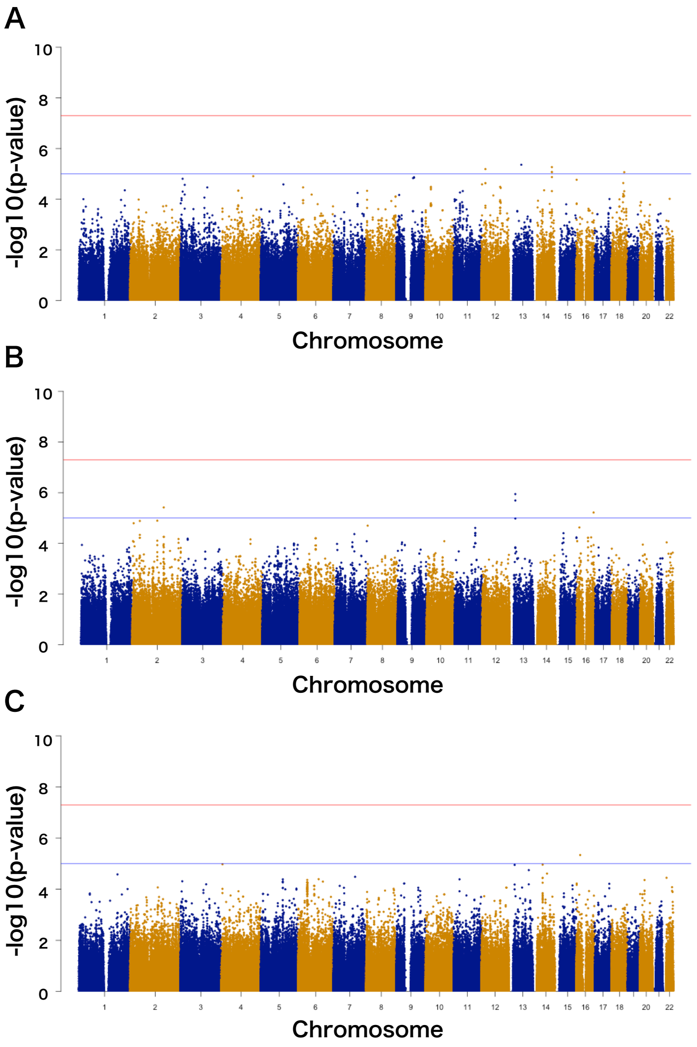

Figure S2. QTL analysis of SPL in schizophrenia (SZ), healthy control (HC) and ALL groups.
Manhattan plots in 60 SZ individuals (A), 166 HC individuals (B) and all 226 individuals (C). Red line and blue line indicate a genome-wide significant p-value ($p=5\times10^{-8}$) and a suggestive p-value ($p=1\times10^{-5}$), respectively.

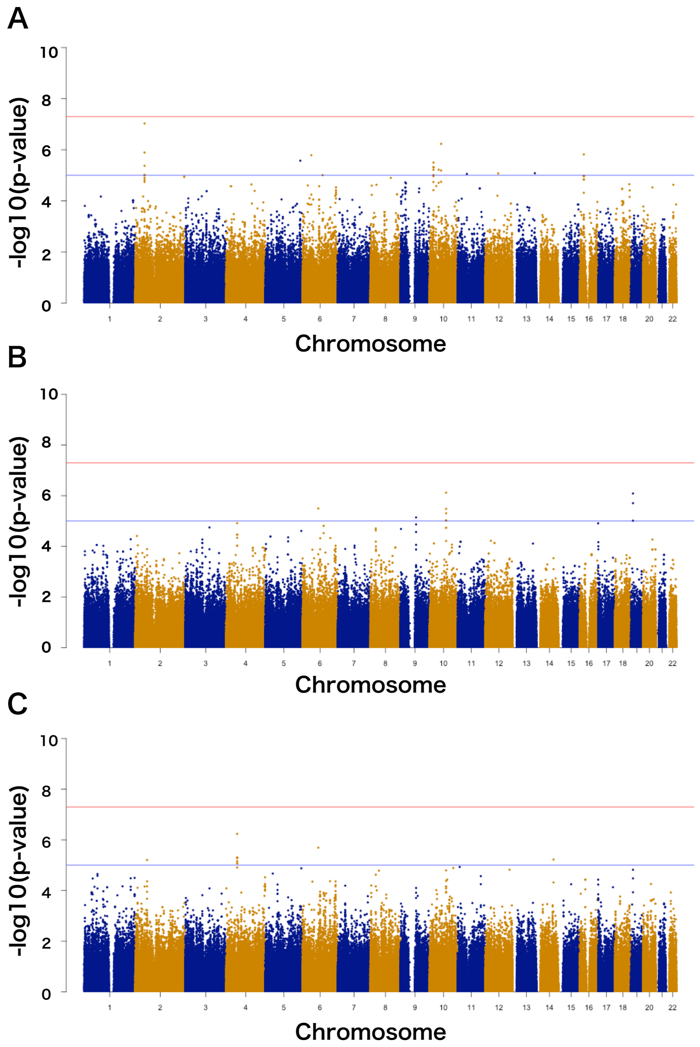

Figure S3. QTL analysis of DF in schizophrenia (SZ), healthy control (HC) and ALL groups.
Manhattan plots in 60 SZ individuals (A), 166 HC individuals (B) and all 226 individuals (C). Red line and blue line indicate a genome-wide significant p-value ($p=5\times10^{-8}$) and a suggestive p-value ($p=1\times10^{-5}$), respectively.

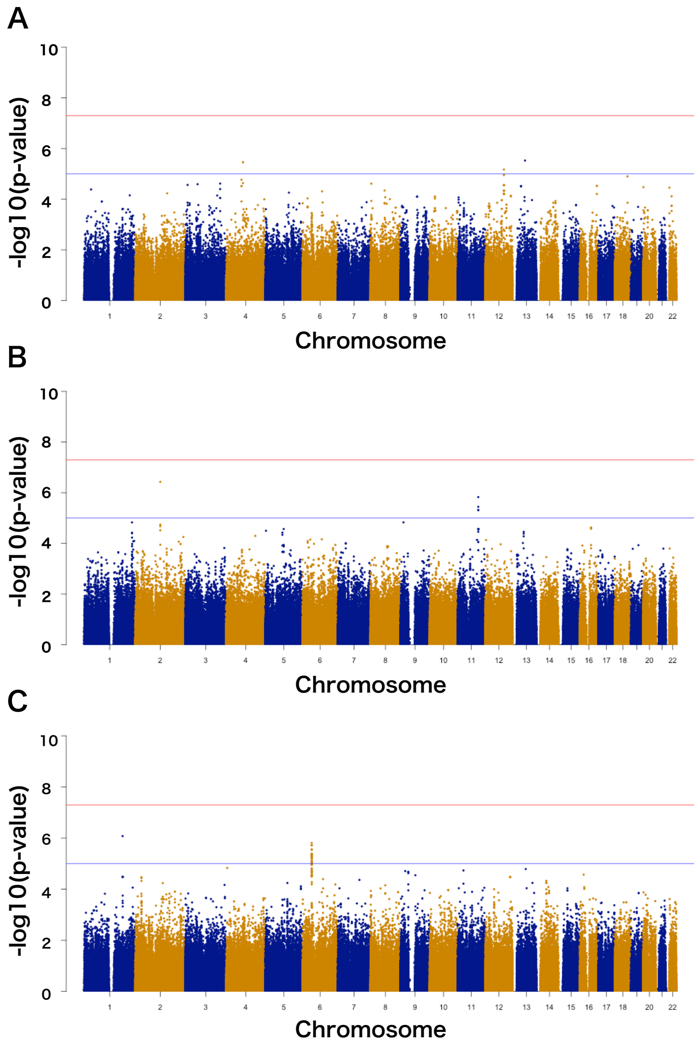

Figure S4. QTL analysis of EMS in schizophrenia (SZ), healthy control (HC) and ALL groups.
Manhattan plots in 60 SZ individuals (A), 166 HC individuals (B) and all 226 individuals (C). Red line and blue line indicate a genome-wide significant p-value ($p=5\times10^{-8}$) and a suggestive p-value ($p=1\times10^{-5}$), respectively.

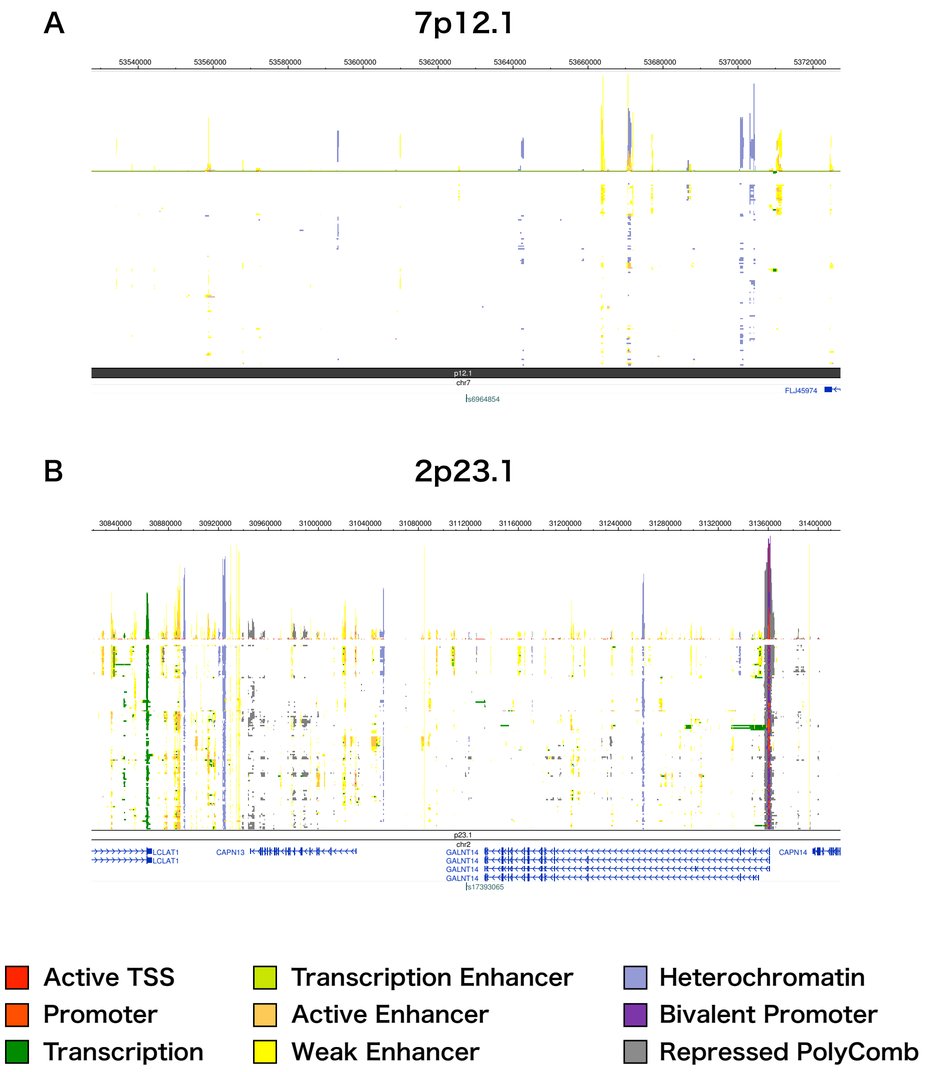

Figure S5. The state model of chromatin in 7p12.1 (A) and 2p23.1(B). The chromatin was segmented into 25 states (i.e. promoter, enhancer, etc.) by ChromHMM algorithm (Ernst et al.) using data provided by NIH Roadmap Epigenomics Consortium (http://www.roadmapepigenomics.org), and was visualized by the WashU Epigenome Browser (http://epigenomegateway.wustl.edu/browser/). Here, 25 states were summarized to 9 states as shown a color legend. The gray shaded stripe represents active/weak enhancer regions.
